# Association of protein intake with the change of lean mass among elderly women: The Osteoporosis Risk Factor and Prevention – Fracture Prevention Study (OSTPRE-FPS)

**DOI:** 10.1017/jns.2015.31

**Published:** 2015-12-16

**Authors:** Masoud Isanejad, Jaakko Mursu, Joonas Sirola, Heikki Kröger, Toni Rikkonen, Marjo Tuppurainen, Arja T. Erkkilä

**Affiliations:** 1 Institute of Public Health and Clinical Nutrition, University of Eastern Finland, PO Box 1627 Kuopio, Finland; 2 Department of Orthopaedics and Traumatology, Kuopio University Hospital, Kuopio, Finland; 3 Kuopio Musculoskeletal Research Unit (KMRU), University of Eastern Finland, Kuopio, Finland; 4 Department of Obstetrics and Gynaecology, Kuopio University Hospital, Kuopio, Finland

**Keywords:** Dietary protein intake, Animal protein intake, Lean mass in elderly, Sarcopenia, aLM, appendicular lean mass, AP, animal protein, BW, body weight, FM, fat mass, LM, lean mass, MPS, muscle protein synthesis, PP, plant protein, TP, total protein

## Abstract

Low protein intake can lead to declined lean mass (LM) in elderly. We examined the associations of total protein (TP), animal protein (AP) and plant protein (PP) intakes with LM. The association of TP intake with LM change was further evaluated according to weight change status. This cross-sectional and prospective cohort study included 554 women aged 68 (sd 1·9) years from the Osteoporosis Risk Factor and Prevention – Fracture Prevention Study (OSTPRE-FPS). The intervention group (*n* 270) received daily cholecalciferol (800 IU; 20 μg) and Ca (1000 mg) for 3 years while the control group received neither supplementation nor placebo (*n* 282). Participants filled out a questionnaire on lifestyle factors and a 3-d food record in 2002 and underwent dual-energy X-ray absorptiometry for body composition measurements at baseline and 3 years. Multiple linear regressions evaluated the association between protein intake and LM, adjusting for relevant covariates. At the baseline TP and AP intakes were positively associated with LM and trunk LM, TP was associated also with appendicular LM (aLM). Follow-up results showed that in the total population and the intervention group, higher TP and AP were associated with increased LM and aLM (*P* ≤ 0·050). No such associations were observed in the control group. PP intake was also associated with aLM change in the total population. Overall, the associations were independent of fat mass. Further, among weight maintainers, TP intake was positively associated with LM, aLM and trunk LM changes (*P* ≤ 0·020). In conclusion, dietary TP, especially AP, intake may be a modifiable risk factor for sarcopenia by preserving LM in the elderly.

The sarcopenic phenotype is characterised by an absolute or relative reduction in lean mass (LM) which can lead to increased risk of fractures, frailty and loss of independence^(^^1^^,^^2^^)^. Older adults over the age of 50 years lose approximately 1–2 % of LM per year^(^^3^^)^. However, the aetiology of LM loss is multifactorial. Dietary protein intake has been considered as one potential contributor to LM change which can determine the balance between protein synthesis and the protein breakdown rate in muscles^(^^2^^,^^4^^)^. Current evidence suggests that age-related loss of LM may be halted or even reversed by increased daily protein intake^(^^5^^–^^7^^)^. The quality of protein intake additionally may play a role in determining the LM. Putatively, animal protein (AP) provides more essential amino acids in comparison with plant protein (PP) sources which can stimulate muscle protein synthesis (MPS)^(^^8^^–^^10^^)^. Vitamin D supplementation further might affect LM directly through different mechanisms. It has been suggested also that vitamin D supplementation might have a synergic relationship with dietary protein intake in increasing LM^(^^11^^–^^15^^)^. However, little is known regarding the interaction between vitamin D supplementation and dietary protein intake and LM and further studies are warranted.

Although data to support guideline for weight-loss treatment in elderly are limited, one of the main targets was the preservation of LM by adequate protein intake^(^^16^^)^. It is well known that dietary protein intake may affect LM and fat mass (FM) partitioning during weight loss^(^^17^^)^. Thus, evaluating the protein intake association with body composition during weight changes may have important implications among elderly who tend to lose weight.

The primary objective of the present study was to examine the associations of total protein (TP), AP and PP intakes with LM at baseline and changes over a 3-year follow-up among elderly women. A secondary objective was to evaluate the association of TP with change of LM according to weight-change status.

## Subjects and methods

### Study population

Data of the present study were collected from the Osteoporosis Risk Factor and Prevention – Fracture Prevention Study (OSTPRE-FPS), which was a 3-year intervention to investigate the effect of Ca and vitamin D supplementation on incidence of falls and fractures among elderly women. The subjects were drawn from the population-based OSTPRE cohort^(^^18^^)^. In total 3432 women volunteered to participate in the study, and 750 women were further randomly invited into this subsample for participating in detailed examinations including the measurement of body composition, and several clinical, physical and laboratory tests^(^^19^^)^. Of these, 554 returned valid food records and had valid body composition measurements for both the baseline and at the 3-year follow-up. The intervention group (*n* 270) received daily cholecalciferol (800 IU; 20 μg) and Ca (1000 mg) for 3 years while the control group received neither supplementation nor placebo (*n* 282)^(^^18^^)^. All participants provided written permission for participation. The study was approved in October 2001 by the ethical committee of Kuopio University Hospital. The study was registered in Clinical trials.gov by the identification NCT00592917^(^^18^^)^.

### Body composition measurements

Height and weight of participants were measured in light indoor clothing without shoes, and BMI was calculated (kg/m^2^). To measure body composition, whole-body dual-energy X-ray absorptiometry scans were performed by specially trained nurses, using the same Lunar Prodigy adhering to the imaging and analysis protocols provided by the manufacturer (Lunar Co.)^(^^20^^,^^21^^)^. Appendicular LM (aLM) was calculated as the sum of the non-fat, non-bone skeletal muscle mass in arms and legs. Further, absolute changes in LM, aLM and trunk LM were calculated by subtracting the baseline values from those measured at year 3.

### Dietary intakes

Dietary intake was collected by using 3-d food records at baseline. A questionnaire and instructions were sent to participants beforehand, and they were returned on the visiting day. The questionnaire was for three consecutive days, including 2 d during the week and 1 d in the weekend (Saturday or Sunday). In the case of uncertainties in the food record, a nutritionist called the participant for additional information^(^^22^^)^. To assess the under-reporting the energy intake:estimated BMR ratio was calculated based on body weight (BW) according to equations given by the Department of Health in the UK^(^^23^^)^. The energy intake:BMR cut-off value for under-reporting was chosen to be 1·49, as derived from Goldberg *et al*.^(^^24^^)^ and Black^(^^25^^)^ and none of the participants was excluded from the analyses. Nutritional intake from food was calculated using the Nutrica program (version 2.5; Finnish social insurance institute, Turku, Finland). Collected data provided calculations of AP (including eggs, dairy products, poultry and meat) and PP sources (cereals, vegetables and fruits) of protein in addition to TP intake.

### Potential confounders

All lifestyle-related information was gathered by the self-administered questionnaire. The questionnaire included questions on age, smoking status (never, former and current), alcohol consumption (portions per week), use of hormone therapy (never used and used) and self-reported vitamin D supplementation. Physical activity level was compiled from frequency of exercise (times per week) and mobility status (restricted or non-restricted). Women were classified as passive if they had restricted or no mobility and exercised ≤2 times/week and those with no mobility restriction and who exercised >2 times/week were classified as active.

### Statistical analysis

All statistical analyses were executed using SPSS software version 21 for Windows (IBM Corp.). A result was significant if the *P* value was <0·05. The protein intakes (TP, AP and PP) were adjusted for energy intake utilising the residual method^(^^26^^)^. An advantage of this method is that it provides a measure of protein intake which is independent of total energy intake. Energy-adjusted protein intake (g/d) was modelled as a continuous variable and categorised into quartiles. Protein intake (g/kg BW) was calculated using crude protein intake divided by BW.

Continuous variables were compared across the quartiles of energy-adjusted TP intake using ANOVA and categorical variables using *χ*^2^ tests. Multiple linear regression models were performed to examine the association between protein intake (g/d) as the independent variable with body composition measures as dependent variables, including LM, aLM and trunk LM at the baseline and changes in them over 3 years of follow-up. Follow-up associations of protein intake with changes in LM, aLM and trunk LM over 3 years of follow-up were explored separately between the intervention and control groups. Tests for a linear trend across quartiles of protein intake were conducted by using the median value in each quartile as a continuous variable in the linear regression model.

Model 1 was adjusted for age, height, total energy intake, study group and in longitudinal setting for baseline LM variables. Model 2 was adjusted for variables in model 1 plus smoking, alcohol use, physical activity level and hormone therapy use. Model 3 was adjusted for variables in model 2 plus baseline FM for cross-sectional setting and change of FM in prospective setting in order to determine whether the associations were independent of FM. For the models for AP and PP, the AP and PP intakes were included in the same regression model to adjust for each other.

We also examined the association between energy-adjusted protein intake (g/d) with LM measurements according to weight-change status. Those who lost over 3 % of their weight during the 3 years of follow-up were classified as weight losers, those who gained over 3 % were classified as weight gainers, and those with moderate change as weight maintainers. This 3 % cut-off was selected and applied to exceed the CV for dual-energy X-ray absorptiometry soft tissue mass^(^^5^^,^^27^^)^.

## Results

The participants were 65·3–71·6 years old; mean age was 68·0 (sd 1·9) years (Table 1). The energy intake was 6560 (sd 1556) kJ/d (1567 (sd 371) kcal), and mean total energy-adjusted protein intake was 68·2 g/d. Median TP intake as a percentage of total energy intake and protein (g/kg BW) by quartiles from quartile 1 to quartile 4 were 14·2 % (0·77 g/kg BW), 16·5 % (0·89 g/kg BW), 18·5 % (0·91 g/kg BW) and 20·1 % (1·17 g/kg BW). Women in the first and third quartiles of energy-adjusted protein intake were more likely to use hormone therapy (46 %) as compared with women in the second and fourth quartiles. Total fat intake (g/d) was highest in quartile 4 and energy intake was significantly higher in higher quartiles of protein intake. TP and AP intakes were significantly higher in higher quartiles of protein intake, while no significant association was observed for PP intake.

**Table 1.** Baseline characteristics of participants across quartiles of energy-adjusted total protein intake (g/d) (Mean values and standard deviations; percentages)

	Quartile 1 (<54·73 g/d) (*n* 138)		Quartile 2 (54·73–66·0 g/d) (*n* 139)		Quartile 3 (66–80·3 g/d) (*n* 139)		Quartile 4 (>80·3 g/d) (*n* 138)		
Characteristics	Mean	sd	Mean	sd	Mean	sd	Mean	sd	P*
Demographics									
Age (years)	68·1	1·9	67·9	1·8	67·6	1·7	67·8	1·9	0·078
Weight (kg)	71·2	12·2	73·7	11·9	71·5	11·3	73·43	12·7	0·014
Height (cm)	157·9	5·6	158·4	5·5	159·4	4·8	158·7	5·3	0·139
BMI (kg/m^2^)	27·2	4·6	26·8	3·6	27·8	4·1	28·0	4·2	0·085
Smoking status (%)									0·194
Never	83·5		85·3		79·9		83·2		
Former	9·0		10·3		15·8		13·9		
Current	7·5		4·4		4·3		2·9		
Portions of alcohol/week (*n*)	3·0	0·7	2·9	0·6	3·0	0·6	4·4	0·7	0·081
Physical activity level (%)†									0·660
Passive	39·1		33·8		40·3		39·9		
Active	60·9		66·2		59·7		60·1		
Hormone therapy use (%)									0·008
Never used	54·0		58·7		54·0		58·7		
Used	46·0		41·3		46·0		41·3		
Body composition									
FM (kg)	28·0	9·3	28·3	7·7	29·6	8·7	29·8	8·7	0·211
LM (kg)	39·1	4·3	40·1	3·8	40·3	4·3	41·2	4·6	0·003
aLM (kg)	16·7	1·8	17·0	1·9	17·1	2·0	17·3	2·0	0·027
Trunk LM (kg)	19·5	2·6	19·6	1·9	20·3	2·4	20·4	2·6	0·001
FM:LM ratio	0·70		0·71		0·72		0·72		0·774
Dietary intakes									
Total energy (kJ/d)	5091	1108	6150	1071	6907	1037	8083	1238	0·036
Fat (% energy)	30·8	5·5	31·0	5·4	31·2	5·9	31·1	5·4	<0·001
Fat (g/d)	55·6	9·9	54·1	10·1	51·3	8·9	66·8	17·6	0·005
Carbohydrate (% energy)	50·8	6·3	49·6	5·1	45·4	5·8	49·5	52·7	<0·001
Carbohydrate (g/d)	204·0	51·5	190·5	45·5	187·6	48·0	193·3	47·8	0·028
Protein (% energy)	16·0	3·0	17·0	2·7	18·0	3·0	19·4	3·1	<0·001
Protein (g/d)	47·0	7·7	60·6	3·2	72·7	4·3	92·0	10·5	<0·001
Animal protein (g/d)	24·7	5·9	35·2	2·0	42·5	2·4	54·3	6·7	<0·001
Vegetable protein (g/d)	23·5	4·4	24·0	4·5	24·4	4·0	24·1	4·2	0·451
Protein (g/kg body weight)	0·79	0·24	0·90	0·23	0·96	0·27	1·18	0·29	<0·001

FM, fat mass; LM, lean mass; aLM, appendicular LM.

* ANOVA or *χ*^2^ tests were used to evaluate the distribution.

† Passive: no mobility and exercise ≤2 times/week; active: no mobility restriction and exercise >2 times/week.

Those in the second and fourth quartiles had higher BW as compared with those in the first and third quartiles. LM, aLM and trunk LM were significantly increased with higher protein intake (Table 1). The absolute LM, aLM and trunk LM changes over the 3 years were +0·69, −0·27 and +0·48 %, respectively. Over the 3 years of follow-up, about 24 % of participants lost >3 % of their BW, 27 % of participants gained >3 % of their BW and 49 % were weight-maintainers (within ±3 % of baseline weight). Mean changes in aLM were a decrease of 0·57 (sd 0·95) kg in weight losers and 0·27 (sd 0·85) kg in weight maintainers and an increase of 0·19 (sd 1·2) kg among weight gainers. There were no significant differences in baseline characteristics between intervention and control groups (see Supplementary Table S1).

At baseline in model 3 energy-adjusted TP was positively associated with LM, aLM and trunk LM (*β* ≥ 0·05; *P* ≤ 0·014). AP intake (g/d) was positively associated with LM and trunk LM (*β* ≥ 0·08; *P* ≤ 0·010) (Table 2). AP intake was associated also with aLM in models 1 and 2; however, the association was no longer significant in model 3 after controlling for FM. No significant association was observed for PP intake except a non-significant association with trunk LM (*β* = 0·06; *P* = 0·083). Results were independent of FM. In the quartile analysis of protein intakes at baseline, women in higher quartiles of TP and AP, but not PP, had significantly greater LM, aLM and trunk LM (*P*_trend_ ≤ 0·026) (data not shown).

**Table 2.** Cross-sectional association of protein intake and total lean mass (LM), appendicular LM (aLM) and trunk LM (*n* 554) (*β* Coefficients with their standard errors)

	LM (kg)			aLM (kg)			Trunk LM (kg)		
	*β*	se	*P*	*β*	se	*P*	*β*	se	*P*
Total protein (g/d)									
Model 1*	0·12	0·01	0·001	0·08	0·01	0·017	0·13	0·82	0·001
Model 2†	0·15	0·01	<0·001	0·09	0·08	0·014	0·16	0·81	<0·001
Model 3‡	0·09	0·01	0·006	0·05	0·08	0·014	0·10	0·94	0·002
Animal protein§(g/d)									
Model 1	0·12	0·01	0·001	0·08	0·01	0·020	0·13	0·83	0·001
Model 2	0·15	0·01	<0·001	0·11	0·01	0·003	0·16	0·81	<0·001
Model 3	0·08	0·01	0·010	0·04	0·01	0·163	0·10	0·88	0·003
Plant protein|| (g/d)									
Model 1	0·02	0·03	0·530	0·01	0·01	0·732	0·02	0·22	0·546
Model 2	0·01	0·03	0·726	−0·01	0·01	0·984	0·01	0·22	0·718
Model 3	0·05	0·04	0·100	0·03	0·02	0·290	0·06	0·24	0·083

* Model 1 was adjusted for age, total energy intake and baseline height and study group.

† Model 2 was adjusted for variables in model 1 plus smoking status, alcohol use per week, physical activity level and hormone therapy use.

‡ Model 3 was adjusted for variables in model 2 plus baseline fat mass.

§ Models for animal protein were also adjusted for plant protein intake.

|| Models for plant protein were also adjusted for animal protein intake.

Results for the prospective analysis are presented separately between intervention and control groups as well as the total population in Table 3. The interaction between energy-adjusted TP, AP and PP intake (g/d) and vitamin D and Ca supplementation was not significant (*P* ≥ 0·730). In model 3, in the intervention group energy-adjusted TP and AP but not PP intakes (g/d) were significantly associated with changes in LM and aLM (*β* ≥ 0·22; *P* = 0·001) over 3 years of follow-up. No significant association was observed in the control group except that PP was non-significantly associated with aLM change (*β* = 0·11; *P* = 0·082). In the total population in model 3, TP and AP were positively associated with LM and aLM changes over 3 years of follow-up (*β* ≥ 0·09; *P* ≤ 0·041). TP and AP were non-significantly associated also with trunk LM change (*β* = 0·08; *P* ≤ 0·088). PP intake in the total population was positively associated with aLM change (*β* = 0·09; *P* = 0·035) and non-significantly associated with LM change (*β* = 0·09; *P* ≤ 0·056) over 3 years of follow-up.

**Table 3.** Prospective association of protein intake and changes in total lean mass (LM), appendicular LM (aLM) and trunk LM between intervention and control groups and the total population (*β* Coefficients with their standard errors)

	LM (kg)			aLM (kg)			Trunk LM (kg)		
	*β*	se	*P*	*β*	se	*P*	*β*	se	*P*
Total protein (g/d)									
Intervention group (*n* 270)									
Model 1*	0·19	8·52	0·003	0·20	0·01	0·001	0·09	6·05	0·032
Model 2†	0·27	8·27	<0·001	0·27	0·01	<0·001	0·15	5·95	0·024
Model 3‡	0·22	10·70	0·001	0·24	0·01	0·001	0·10	7·60	0·125
Control group (*n* 282)									
Model 1	−0·03	9·97	0·559	−0·03	0·01	0·539	0·01	5·82	0·955
Model 2	−0·40	9·30	0·538	−0·51	0·01	0·429	0·01	6·05	0·877
Model 3	−0·07	12·80	0·275	−0·05	0·01	0·360	−0·03	8·30	0·548
Total population (*n* 552)									
Model 1	0·07	0·06	0·115	0·06	0·04	0·098	0·05	0·04	0·276
Model 2	0·09	0·06	0·035	0·08	0·04	0·050	0·08	0·04	0·075
Model 3	0·10	0·01	0·032	0·09	0·04	0·041	0·08	0·04	0·081
Animal protein§(g/d)									
Intervention group (*n* 270)									
Model 1	0·17	8·53	0·004	0·19	0·01	0·001	0·08	6·08	0·068
Model 2	0·27	8·26	<0·001	0·27	0·01	<0·001	0·14	6·95	0·027
Model 3	0·22	10·40	0·001	0·24	0·01	0·001	0·09	7·10	0·151
Control group (*n* 282)									
Model 1	−0·03	8·96	0·557	−0·04	0·01	0·528	0·01	5·90	0·952
Model 2	−0·04	9·28	0·539	−0·05	0·01	0·430	0·10	6·05	0·874
Model 3	−1·03	11·90	0·301	−0·06	0·07	0·334	−0·03	7·70	0·625
Total population (*n* 552)									
Model 1	0·06	0·06	0·142	0·07	0·04	0·092	0·05	0·04	0·287
Model 2	0·09	0·06	0·049	0·08	0·04	0·047	0·08	0·04	0·080
Model 3	0·10	0·06	0·037	0·09	0·04	0·037	0·08	0·04	0·088
Plant protein|| (g/d)									
Intervention group (*n* 270)									
Model 1	0·09	22·12	0·124	0·09	0·01	0·098	0·05	15·79	0·430
Model 2	0·05	22·01	0·436	0·03	0·01	0·608	0·03	15·84	0·575
Model 3	0·10	0·02	0·137	0·08	0·01	0·182	0·06	0·15	0·360
Control group (*n* 282)									
Model 1	0·06	24·86	0·323	0·09	0·01	0·126	0·01	16·35	0·935
Model 2	0·09	26·57	0·160	0·11	0·01	0·090	0·03	17·33	0·597
Model 3	0·09	0·02	0·158	0·11	0·01	0·082	0·03	0·17	0·601
Total population (*n* 552)									
Model 1	−0·81	0·36	0·223	−0·15	0·21	0·019	−0·04	2·4	0·482
Model 2	0·09	0·17	0·063	0·09	0·01	0·039	0·04	1·11	0·327
Model 3	0·09	0·17	0·056	0·09	0·01	0·035	0·04	1·11	0·334

* Model 1 was adjusted for age, total energy intake and baseline height and study group.

† Model 2 was adjusted for variables in model 1 plus smoking status, alcohol use per week, physical activity level and hormone therapy use.

‡ Model 3 was adjusted for variables in model 2 plus baseline fat mass.

§ Models for animal protein were also adjusted for plant protein intake.

|| Models for plant protein were also adjusted for animal protein intake.

In a follow-up analysis using quartiles of protein intakes in the intervention group, women in the highest quartiles of TP and AP intakes had significantly increased LM and aLM (*P*_trend_ *≤* 0·001) as compared with those in the lower quartiles, while no such association was observed for PP intake. No significant association was observed in the control group. Further, in the total population, a non-significant association was observed between higher quartiles of TP and aLM change (*P*_trend_ = 0·079) and PP intake was significantly associated with less decline in aLM (*P*_trend_ = 0·027) (data not shown).

The association of energy-adjusted TP (g/d) with LM changes was further evaluated according to weight-change status. Weight change and energy-adjusted TP interactions were significant (*P*_interaction_ < 0·001). Among weight maintainers, energy-adjusted TP (g/d) was associated with change in LM and aLM and trunk LM (*β* ≥ 0·13; *P* ≤ 0·020) (Table 4).

**Table 4.** Association of total protein intake (g/d) and changes of lean mass (LM), appendicular LM and trunk LM by weight change status (*n* 551) (*β* Coefficients with their standard errors)

		LM (kg)			aLM (kg)			Trunk LM (kg)		
	*n*	*β*	se	P*	*β*	se	*P*	*β*	se	*P*
Weight losers†	180	0·10	0·01	0·237	0·11	0·01	0·150	0·03	0·01	0·704
Weight maintainers	278	0·29	0·07	0·001	0·13	0·01	0·020	0·17	0·01	0·005
Weight gainers	96	−0·11	0·02	0·321	−0·27	0·01	0·783	−0·09	0·01	0·445

* Adjusted for age, total energy intake, baseline LM, aLM and trunk LM, height, smoking status, alcohol portions per week, physical activity level, hormone therapy use and study group.

† Those who lost over 3 % of their baseline weight during the 3 years of follow-up were classified as weight losers, those who gained over 3 % as weight gainers, and those with moderate change as weight maintainers.

## Discussion

The primary findings of this study were that at baseline higher energy-adjusted TP and AP intakes were positively associated with LM and trunk LM, and that TP intake was also associated with greater aLM. Follow-up results showed that in the intervention group as well as the total population higher TP and AP intakes were positively associated with changes in LM and aLM over 3 years of follow-up, while no significant association was observed in the control group. No such association was observed for PP intake except that in the total population PP intake was significantly associated with less decline in aLM over 3 years of follow-up. These associations remained significant even after adjusting for FM. Further, among weight maintainers TP intake was positively associated with LM, aLM and trunk LM changes.

Houston *et al*. showed that among women aged 70–79 years (*n* 2066), those with higher protein intake (19 % of total energy intake) lost 40 % less LM as compared with those with lower intake (11 % of total energy intake) over a 3-year follow-up^(^^5^^)^. Similarly, Meng *et al*. found that elderly women with higher TP intake (average >1·6 g/kg BW or 20·0 % of energy) had higher LM as compared with those with lower protein intake (average 0·85 g/kg BW or 18·0 % of energy)^(^^7^^)^. The results from the present study were consistent with those previous studies suggesting that higher protein intake is beneficial to LM^(^^5^^–^^8^^,^^28^^)^.

For older people (>65 years) to maintain and regain muscle mass and function, an average daily intake at least in the range of 1·0 to 1·2 g/kg BW is recommended^(^^29^^,^^30^^)^, which is higher than the current RDA (0·8 g/kg BW)^(^^31^^)^. A preponderance of evidence now suggests that ageing might result in the stimulation of MPS becoming resistant to the anabolic effect of hyperaminoacidaemia, particularly at lower protein intakes^(^^10^^)^. The decreased MPS might partially be explained by decreased mammalian target of rapamycin and the 70-kDa ribosomal protein S6 kinase signalling^(^^32^^)^, and changes in positive regulators like insulin-like growth factor 1 and negative regulators (e.g. adenosine monophosphate–activated protein kinase) of this pathway^(^^33^^,^^34^^)^. AP contains essential amino acids, which trigger the aforementioned signalling pathways, enhancing protein accretion and LM^(^^34^^)^.

Only a few studies have examined the effect of protein source on body composition in older adults^(^^5^^,^^6^^,^^9^^)^. In a study by Sahni *et al*. in men and women aged 59 (sd 9) years TP intake was 80 (sd 27) g/d in men and 76 (sd 26) g/d in women. In men and women, leg LM was higher in participants in the highest quartile of TP and AP intake compared with those in the lowest quartiles of intake^(^^6^^)^. PP intake was not associated with LM in either sex. Although plant-based diets are low in certain essential amino acids, they have been linked with higher LM^(^^17^^,^^35^^)^. Our data suggest accordingly that AP but not PP was associated with greater LM; however, PP was associated with increased aLM in the total population over 3 years of follow-up. Thus, the dietary protein quality (AP *v.* PP) intake in relation to health outcomes and LM needs to be further clarified.

Vitamin D can potentially affect LM through different mechanisms which are yet not fully elucidated^(^^14^^,^^15^^)^. It has been suggested that vitamin D deficiency is linked with muscle weakness. The presence of vitamin D receptor in muscle tissue cells is yet a matter of debate^(^^13^^,^^36^^)^. Previous findings regarding the effect of vitamin D supplementation on LM are inconclusive^(^^11^^,^^12^^)^. A recent meta-analysis suggested that vitamin D has no significant effect on LM^(^^11^^)^. A separate investigation in the present data showed no significant effect of vitamin D (800 IU; 20 μg) and Ca supplementation on LM (M Isanejad, J Sirola, H Kröger and T Rikkonen, unpublished results). Furthermore, some evidence suggests that vitamin D and protein intake might have a synergic effect on increasing LM^(^^15^^,^^37^^)^. LM loss during ageing my partially be explained by the decreased ability of muscle to respond to anabolic stimuli provided by dietary protein through decreased MPS to physiological concentrations of amino acids and insulin^(^^38^^)^. Of particular interest, vitamin D deficiency was associated with insulin resistance *in vivo*^(^^39^^)^, while vitamin D treatments have been linked to an increased expression of insulin receptor in skeletal muscle^(^^15^^,^^40^^)^. The present study showed that the association of TP and AP with changes in LM and aLM was stronger in those women who received the vitamin D and Ca supplementation. Although there were no differences in the baseline characteristics between the intervention and control groups, it is possible that there were other modifying factors. To the best of our knowledge this was the first cohort study to evaluate the interaction between vitamin D and Ca supplementation and protein intake with LM and further studies are warranted.

Although data to support guidelines for weight loss in the elderly are limited, one of the main targets is the preservation of LM by adequate protein intake^(^^16^^)^. Previously an intervention study has shown that a diet with high protein intake (35 % of energy) was associated with preservation of LM during weight loss^(^^41^^)^. Our data suggest that associations of TP intake and LM, aLM and trunk LM were significant in weight maintainers when weight changes do not confound. Therefore, these findings suggest that it is worth paying attention to the role of dietary protein intake in weight change among the ageing population.

The strength of the present study was that all the body composition measurements were available at baseline and over a 3-year period. We performed a careful adjustment for potential known confounders; however, there might be other factors that were not captured in this study. To adjust for body size as an important modifying factor for LM, a variety of methods have been used, and we chose baseline height which has been applied and used before^(^^5^^,^^7^^,^^42^^)^. Worthy of note is that aLM provides a measure in which the component of muscle is relatively large.

A limitation of this study was that the study population consists of only elderly women and therefore caution should be taken when generalising the findings to elderly men. However, in previous studies when exploring associations between protein intake and LM, significant associations were observed similarly for men and women^(^^5^^,^^6^^)^. It would be beneficial for future studies to explore the association of protein intake and LM change in both males and females. The 3-d dietary records method has been described as a suitable instrument for assessing energy and protein intake in elderly people^(^^43^^,^^44^^)^, which has been also used and applied to measure AP and PP intake^(^^8^^)^. The latter study has also been validated against urinary nitrogen studies in both community-dwelling and institutionalised elderly people^(^^44^^)^. However, errors in recording and change in dietary intake as well as type of protein intake are not avoidable, but the distribution of errors is unlikely to be related to the outcome. The dietary intake assessment was obtained only at baseline which may be insufficient to capture long-term dietary exposures. Information of intentionality of weight loss during the 3 years of follow-up was not available; therefore, it might be possible that those who lost weight over this period had generally lower inferior health condition as compared with weight maintainers or weight gainers. Lastly, causal associations cannot be obtained due to the observational nature of this study.

In conclusion, our findings support the current evidence that higher TP and in particular AP intakes are beneficial in preserving LM. A remarkable finding of this study was that the associations of TP, AP with increased LM were more apparent among elderly women who maintained their weight and received vitamin D and Ca supplementation. Since dietary protein intake, vitamin D and weight change are important health concerns of ageing, our results might underscore an important message for public health.

## Supplementary material

To view supplementary material for this article, please visit http://dx.doi.org/10.1017/jns.2015.31
